# Supplementary material for: Nucleotide exchange is sufficient for Hsp90 functions in vivo
Source: Nat Commun. 2023 Apr 29;14:2489. doi: 10.1038/s41467-023-38230-0 (PMC10148809; doi:10.1038/s41467-023-38230-0)
Supplement: Supplementary file 1 — Supplementary Information [file 41467_2023_38230_MOESM1_ESM.pdf]

Nucleotide exchange is sufficient for Hsp90 functions in vivo

Supplementary Figures

Supplementary Figure 1

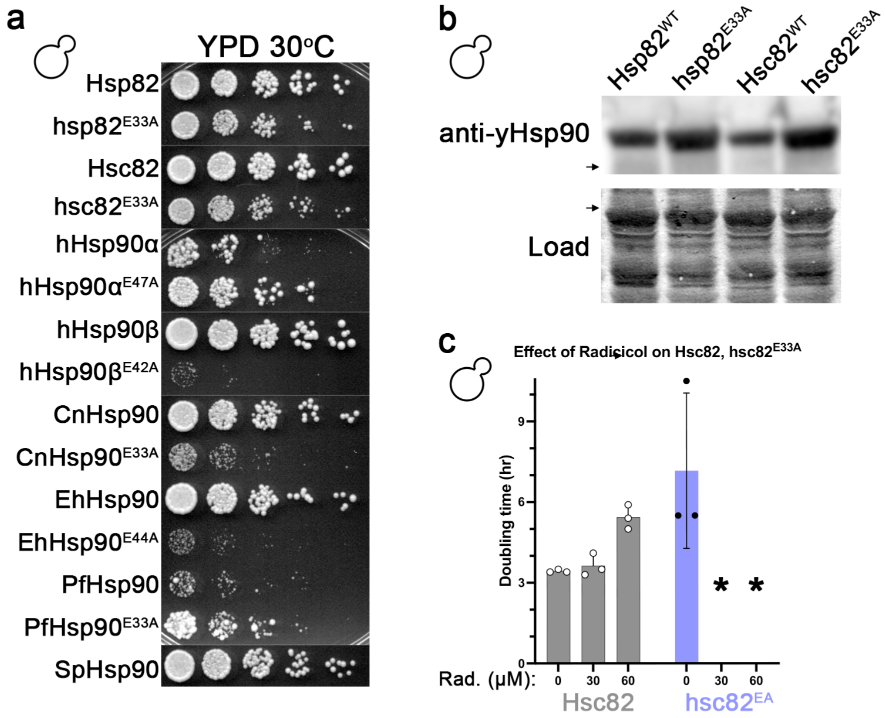

Supplementary Figure 1. The effect of EA mutation in various Hsp90 orthologs on growth of S. cerevisiae. A. *S. cerevisiae* cells expressing the indicated Hsp90s were picked from FOA plates and spotted on to YPD plates as described in the Methods. **B.** Western blot showing the relative expression of the indicated Hsp90s in log-phase *S. cerevisiae* cells. “Load” is a portion of the stained membrane showing equal loading of cell lysates. **C.** Cells expressing the Hsc82-E33A variant were hypersensitive to radicicol. Bars are the average doubling times of three biological replicates (circles) and the error bars are the standard deviation. Asterisks denote conditions where no growth was observed.

Supplementary Figure 2

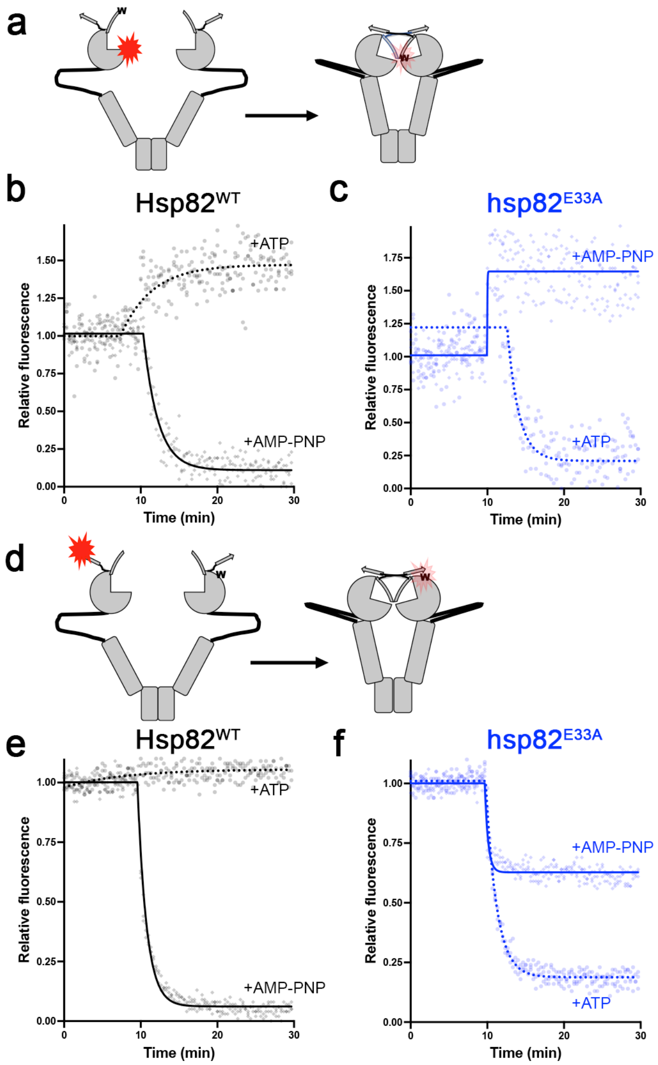

Supplementary Figure 2. EA flips the response to nucleotides in lid closing and β-strand swapping PET. A. Cartoon depicting the lid repositioning PET experiment. **B**. AMP-PNP (Black diamonds are the data points and solid line is the fit) but not ATP (black circles and dotted line) induced lid repositioning in wild type Hsp82. **C**. ATP (blue circles and dotted line) but not AMP-PNP (blue diamonds and solid line) induced lid repositioning in Hsp82-EA. **D**. Cartoon depicting the β-strand swap PET experiment. **E**. AMP-PNP (Black diamonds and solid line) but not ATP (black circles and dotted line) induced β-strand swapping in wild type Hsp82. **F**. ATP (blue circles and dotted line) but not AMP-PNP (blue diamonds solid line) induced β-strand swapping in Hsp82-EA. Nucleotides were added at T=10. For all PET, lines are the exponential decays fits of the average of three independent experiments (raw data shown). Source data are provided as a Source Data file.

Supplementary Figure 3

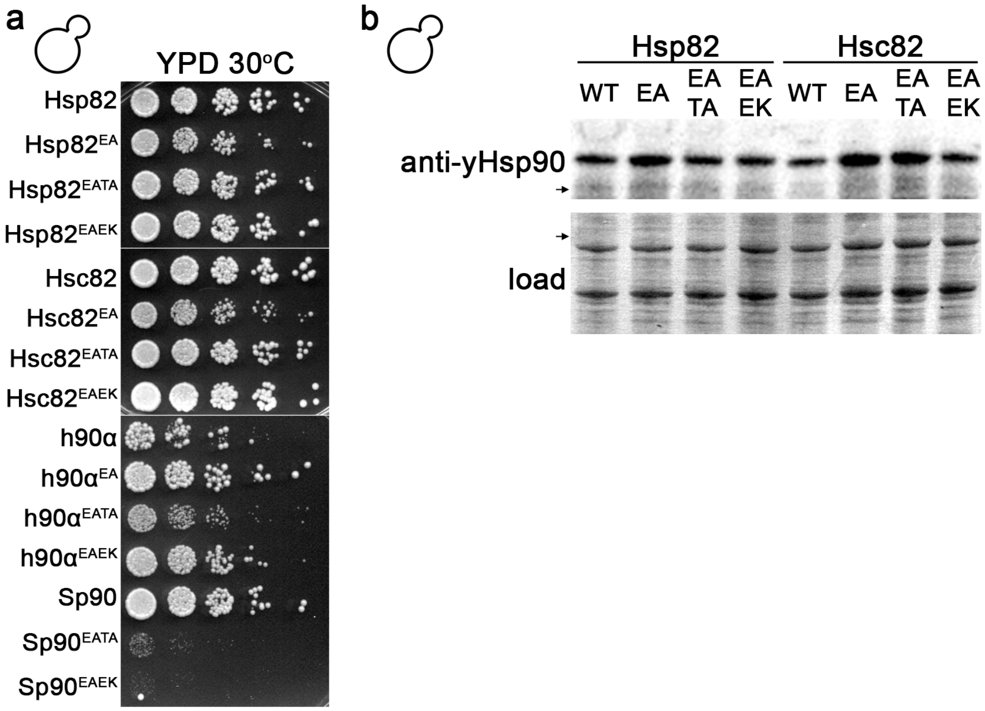

Supplementary Figure 3. The effect of the TA and EK suppressors on EA-mediated growth effects in different Hsp90s. A. Growth of *S. cerevisiae* cells expressing the indicated Hsp90s on right. **B.** Representative western blot (of three independent experiments) showing the expression of the indicated Hsp82 (left lanes) or Hsc82 (right lanes) variant. Load is a portion of the blotted membrane stained with amido black. Arrows denote the location of the 75 kD molecular weight marker.

Supplementary Figure 4

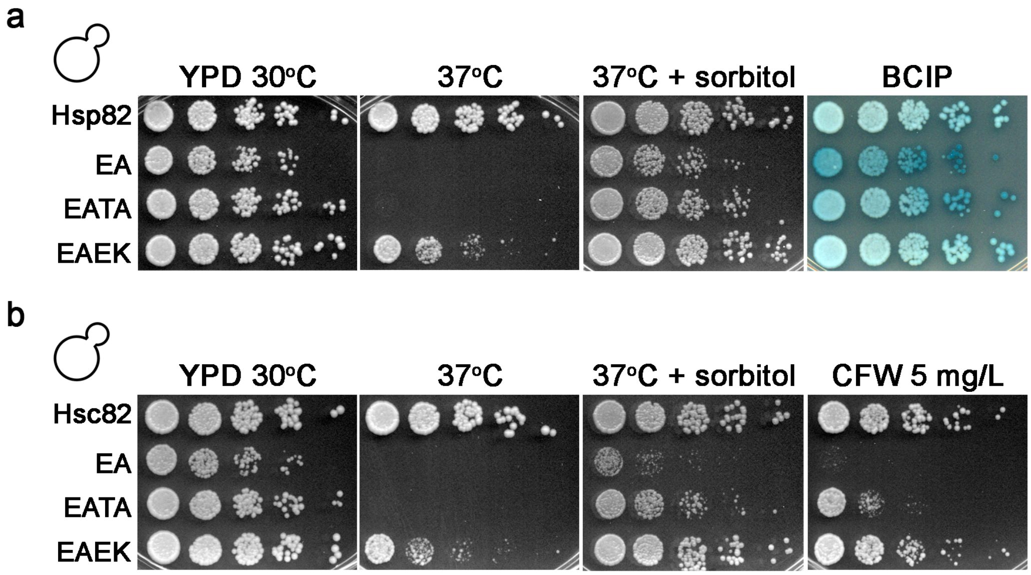

Supplementary Figure 4. The effect of TA and EK on EA-mediated CWI defects. A. *S. cerevisiae* cells expressing Hsp82-EAEK grew better at 37°C than cells expressing Hsp82-EA. Cells expressing Hsp82-EATA or -EAEK had less leaky cell walls than Hsp82-EA cells. **B.** *S. cerevisiae* cells expressing Hsc82-EATA or -EAEK were less sensitive to CFW than cells expressing Hsc82-EA. Cells expressing Hsc82-EAEK were less temperature sensitive than Hsp82-EA cells.

Supplementary Figure 5

a

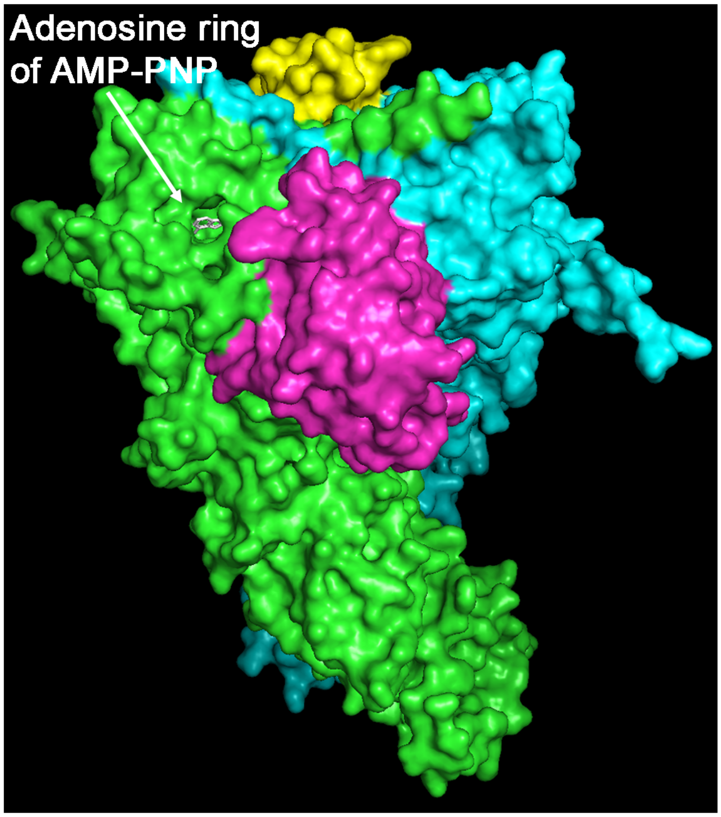

b

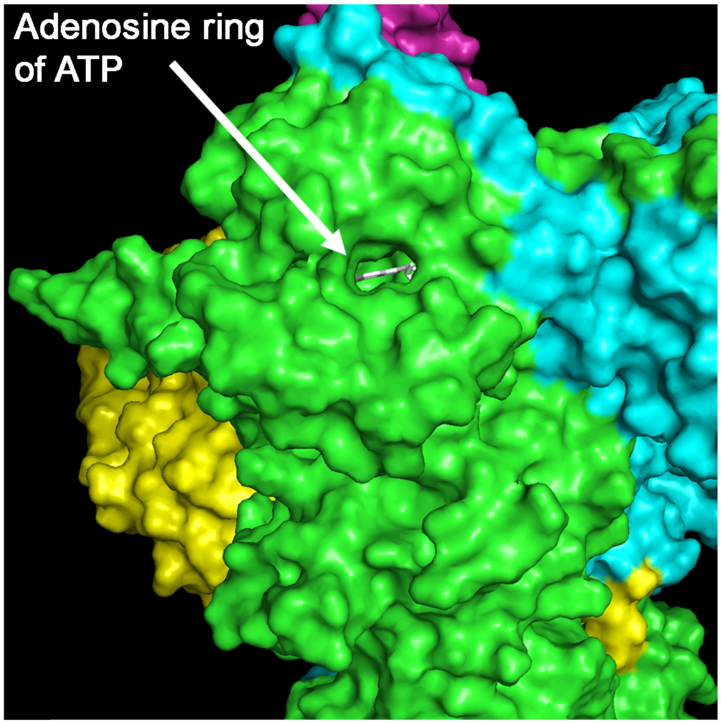

Supplementary Figure 5. In the closed clamp a channel from the surface to the nucleotide is observed. A. Surface view of PDB ID 2cg9, the closed-clamp Hsp82^{A107N} (green and cyan) structure in complex with Sba1 (magenta and yellow). The adenosine ring of the bound nucleotide (white) can be readily observed through a channel in the N-terminal domain of Hsp82. The outer rim of the channel is comprised of residues K44, I82, E88, N92, K102 and A103. The channel in the cyan protomer is on the reverse side of the molecule as displayed. **B.** The analogous channel in hHsp90β (PBD ID 5fwk). Green and cyan are the hHsp90β protomers, magenta is Cdc37, yellow is Cdk4 and white is ATP.

Supplementary Table 1. Oligonucleotides used in this study. Introduced restriction sites are underlined. “F” denotes forward cloning primer, “R” denotes reverse cloning primer, all others are mutagenic primers.

Purpose	Sequence (5’->3’)
HSC82 -500 bp F	GATCGGATCCTTGAGTACAACTACGATAAG
HSC82 +500 bp R	GATCGGATCCGGCGGAATTGGTATGCTATTC
SpHsp90 ORF F	TAGTTTCGACGGATTCTAGAACTAGTATGTCGAACACAGAAACTTTC
SpHsp90 ORF R	TGACATAACTAATTACATGAGTCGACTTAATCGACTTCCTCCATC
SpHsp90 -669 F	GATCGGATCCGATACAGTTAAACACTTTAAATG
SpHsp90 +316 R	GATCGGATCCGCTCTCACATGACTAGAAAA
tdh1^{+} promoter F	GATCGAGCTCTAAAGTATGGAAAATCAAAATTCC
tdh1^{+} promoter R	GATCACTAGTTTTGAATCAAGTGTAAATCAATAC
E33A Hsp82	CAAGGAAATTTTCTTGAGAGCTCTGATATCTAATGCCTCGGA
E33A Hsc82	CAAGGAAATTTTCTTGAGAGCTCTGATCTCTAACGCCTCCGA
E47A hHsp90α	CAAAGAGATCTTTCTGAGAGCTCTCATTTCAAATTCATCAGA
E42A hHsp90β	CAAGGAGATTTTCCTTCGGGCTTTGATCTCTAATGCTTCTGA
E33A CnHsp90	TAAGGAAATATTCCTTAGGGCTCTAATCTCTAACAGTTCAGA
E44A EhHsp90	TAAGGATATATTCCTGAGGGCTCTGATTTCTAACTGCAGCGA
E33A PfHsp90	CAAAGAAATATTTCTTAGGGCTCTAATTTCAAATGCAAGTGA
E34A SpHsp90	CAAGGAAATTTTCCTTCGTGCTCTTATTTCCAACGCTTCCGA
D79N Hsp82	AAAAAGTTTTGGAAATCAGAAATTCTGGTATTGGTATGACC
D79N Hsc82	AAAAAGTTTTGGAAATCAGAAATTCTGGTATTGGTATGACC
D93N hHsp90α	ATCGAACTCTCACTATTGTGAATACTGGAATTGGAATGACC
D88N hHsp90β	AACGTACCCTGACTTTGGTAAATACAGGCATTGGCATGACCAA
D79N CnHsp90	AAGGGACCTTGACTATAAGGAATACGGGGATAGGTATGACGAA
D90N EhHsp90	ATAAACAGCTTATTATTGAGAATACGGGGATTGGGATGACCAA
D79N PfHsp90	ATAACACCCTGACGATAGAGAATAGCGGGATCGGAATGACC
D80N SpHsp90	ACAAAATCCTTAGCATTCGCAATACCGGTATTGGTATGACC
T171A Hsp82	ATGAAAGAATTGGTAGGGGTGCTATCTTGAGGTTATTCTTGAA
E372K Hsp82	TCAAGGGTGTTGTTGACTCTAAAGATTTACCATTGAATTTGTC
T171A Hsc82	ACGAAAGAATTGGTAGAGGTGCTGTCTTGAGATTATTCTTGAA
E368K Hsc82	TCAAGGGTGTTGTTGACTCTAAAGATTTACCATTGAATTTG
T184A hHsp90α	GTGAACCTATGGGTCGTGGAGCTAAAGTTATCCTACACCTGAA
E392K hHsp90α	TTAGAGGGGTGGTAGACTCGAAAGATCTCCCTCTAAACATATC
T179A hHsp90β	GTGAGCCCATTGGCAGGGGTGCTAAAGTGATCCTCCATCTTAA
E384K hHsp90β	TCCGTGGTGTGGTTGACTCTAAAGATCTGCCCCTGAACATCTC
T172A SpHsp90	GACCTCGCTTGTTACGTGGTGCTGAGATTCGTCTCTTCATGAA
E367K SpHsp90	TTAAGGGTGTTGTTGATTCTAAAGACTTGCCCTTGAACTTGTC
