# Supplementary material for: Nucleotide exchange is sufficient for Hsp90 functions in vivo
Source: Nat Commun. 2023 Apr 29;14:2489. doi: 10.1038/s41467-023-38230-0 (PMC10148809; doi:10.1038/s41467-023-38230-0)
Supplement: Supplementary file 3 — Reporting Summary [file 41467_2023_38230_MOESM3_ESM.pdf]

nature portfolio

Corresponding author(s): Michael Reidy

Last updated by author(s): Mar 27, 2023

Reporting Summary

Nature Portfolio wishes to improve the reproducibility of the work that we publish. This form provides structure for consistency and transparency in reporting. For further information on Nature Portfolio policies, see our Editorial Policies and the Editorial Policy Checklist.

Statistics

For all statistical analyses, confirm that the following items are present in the figure legend, table legend, main text, or Methods section.

n/a	Confirmed	
☐	☒	The exact sample size (*n*) for each experimental group/condition, given as a discrete number and unit of measurement
☐	☒	A statement on whether measurements were taken from distinct samples or whether the same sample was measured repeatedly
☒	☐	The statistical test(s) used AND whether they are one- or two-sided<br>*Only common tests should be described solely by name; describe more complex techniques in the Methods section.*
☒	☐	A description of all covariates tested
☒	☐	A description of any assumptions or corrections, such as tests of normality and adjustment for multiple comparisons
☐	☒	A full description of the statistical parameters including central tendency (e.g. means) or other basic estimates (e.g. regression coefficient) AND variation (e.g. standard deviation) or associated estimates of uncertainty (e.g. confidence intervals)
☒	☐	For null hypothesis testing, the test statistic (e.g. *F*, *t*, *r*) with confidence intervals, effect sizes, degrees of freedom and *P* value noted<br>*Give P values as exact values whenever suitable.*
☒	☐	For Bayesian analysis, information on the choice of priors and Markov chain Monte Carlo settings
☒	☐	For hierarchical and complex designs, identification of the appropriate level for tests and full reporting of outcomes
☒	☐	Estimates of effect sizes (e.g. Cohen's *d*, Pearson's *r*), indicating how they were calculated

Our web collection on statistics for biologists contains articles on many of the points above.

Software and code

Policy information about availability of computer code

Data collection	Data collected using the BMG Omega plate reader (PET, FRET, ATPase and growth curves) was collected using the BMG Omega software version 5.5.
Data analysis	Data was analyzed using Microsoft Excel version 16.54 and/or GraphPad Prism 9

For manuscripts utilizing custom algorithms or software that are central to the research but not yet described in published literature, software must be made available to editors and reviewers. We strongly encourage code deposition in a community repository (e.g. GitHub). See the Nature Portfolio guidelines for submitting code & software for further information.

Data

Policy information about availability of data

All manuscripts must include a data availability statement. This statement should provide the following information, where applicable:

- Accession codes, unique identifiers, or web links for publicly available datasets
- A description of any restrictions on data availability
- For clinical datasets or third party data, please ensure that the statement adheres to our policy

All data generated or analyzed during this study are included in this published article (and its supplementary information files). Images of protein structures were sourced from Protein Database files 2cg9 and 5fwk.

Human research participants

Policy information about studies involving human research participants and Sex and Gender in Research.

Reporting on sex and gender	n/a
Population characteristics	n/a
Recruitment	n/a
Ethics oversight	n/a

Note that full information on the approval of the study protocol must also be provided in the manuscript.

Field-specific reporting

Please select the one below that is the best fit for your research. If you are not sure, read the appropriate sections before making your selection.

☒ Life sciences ☐ Behavioural & social sciences ☐ Ecological, evolutionary & environmental sciences

For a reference copy of the document with all sections, see nature.com/documents/nr-reporting-summary-flat.pdf

Life sciences study design

All studies must disclose on these points even when the disclosure is negative.

Sample size	Sample sizes for S. cerevisiae plasmid shuffles were at least three independent transformants. Sample sizes for Sz. pombe plasmid shuffles were at least five independent transformants. These sample sizes for these types of experiments are common in the yeast field. We chose these many transformants as each represents a biological replicate, and generally three to five biological replicates are sufficient to observe the true phenotype associated with given allele. Each experiment was performed at least three times.
Data exclusions	No data was excluded.
Replication	Plasmid shuffle and biochemical experiments had at least three independent replicates.
Randomization	No randomization was required because we did not have large datasets or trials.
Blinding	Blinding was not required for the type of experiments we present.

Reporting for specific materials, systems and methods

We require information from authors about some types of materials, experimental systems and methods used in many studies. Here, indicate whether each material, system or method listed is relevant to your study. If you are not sure if a list item applies to your research, read the appropriate section before selecting a response.

Materials & experimental systems

n/a	Involved in the study	
☐	☒	Antibodies
☒	☐	Eukaryotic cell lines
☒	☐	Palaeontology and archaeology
☒	☐	Animals and other organisms
☒	☐	Clinical data
☒	☐	Dual use research of concern

Methods

n/a	Involved in the study	
☒	☐	ChIP-seq
☒	☐	Flow cytometry
☒	☐	MRI-based neuroimaging

Antibodies

Antibodies used	anti-Hsc82 (Abcam ab30920); anti-Mpk1 E-9 (Santa Cruz Biotech sc-133189); anti-P-p44/42 MAPK (Cell Signalling Technology 4370S)
Validation	Antibodies were validated by us using appropriate S. cerevisiae strains that had been treated under appropriate conditions or were deleted for an appropriate gene, compared to untreated/wild type controls. anti-Hsc82 recognized a single band ~82 kD in size from lysates of cells expressing yeast, but not Human, Hsp90. anti-Mpk1 E-9 recognized a single band ~55 kD in size from lysates of wild

type yeast cells, but no band in slt2 deletion cells. anti-P-p44/42 MAPK recognized a single band ~ 55 kD in size from lysates of wild type yeast, the same band but much more intense in wild type cells treated with calofluor white, and no band in slt2 deletion cells.
