# Supplementary figures and images for: Nucleotide exchange is sufficient for Hsp90 functions in vivo
Source: Nat Commun. 2023 Apr 29;14:2489. doi: 10.1038/s41467-023-38230-0 (PMC10148809; doi:10.1038/s41467-023-38230-0)

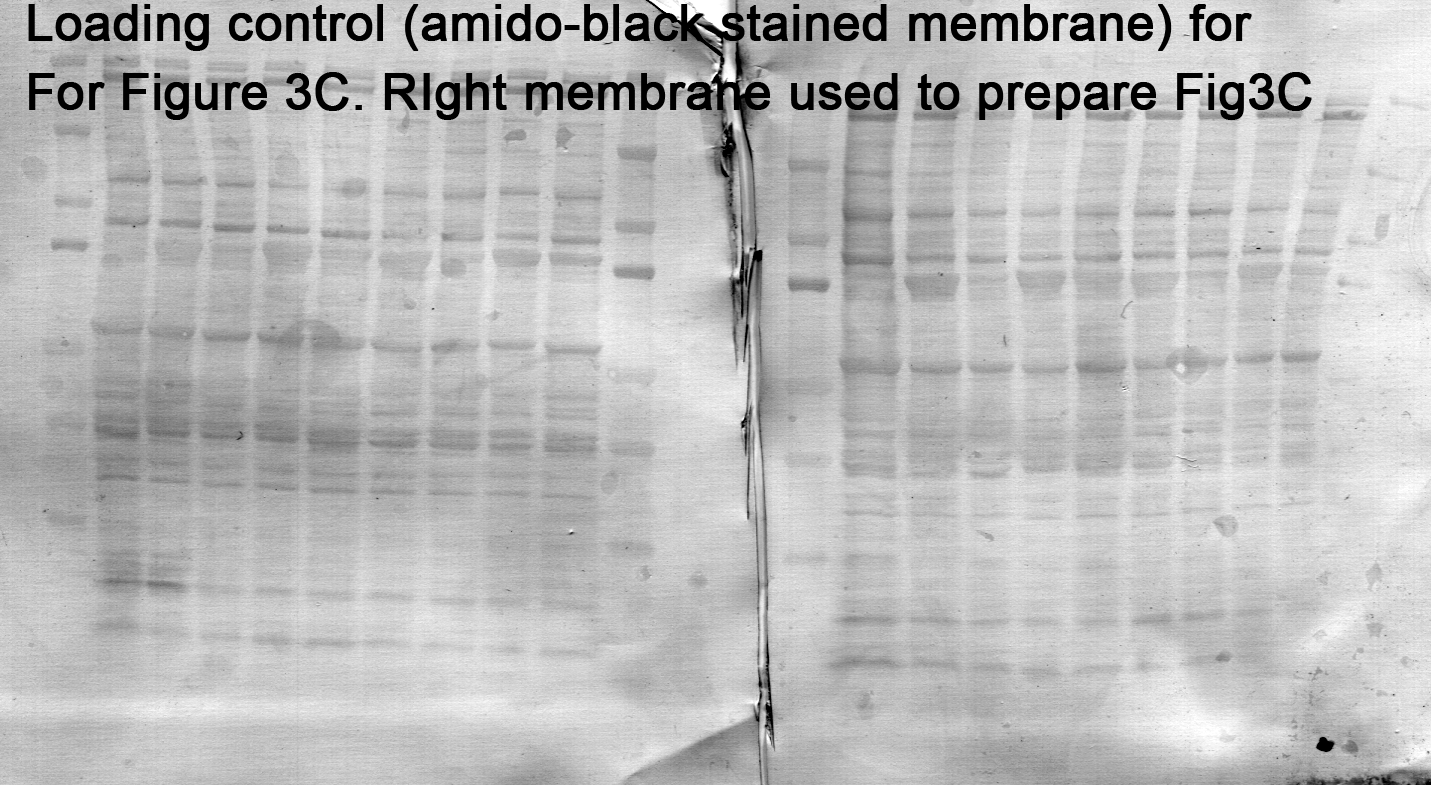

Supplement: Supplementary file 4 — Source Data [file 41467_2023_38230_MOESM4_ESM.zip › Source Data files/Uncropped blots/Uncropped loading control Fig3C.tif]

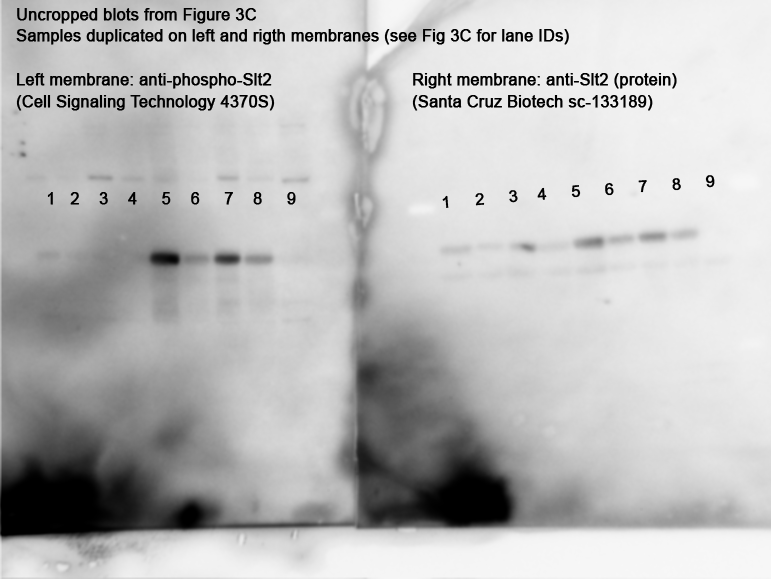

Supplement: Supplementary file 4 — Source Data [file 41467_2023_38230_MOESM4_ESM.zip › Source Data files/Uncropped blots/Uncropped blot Fig3C.tif]

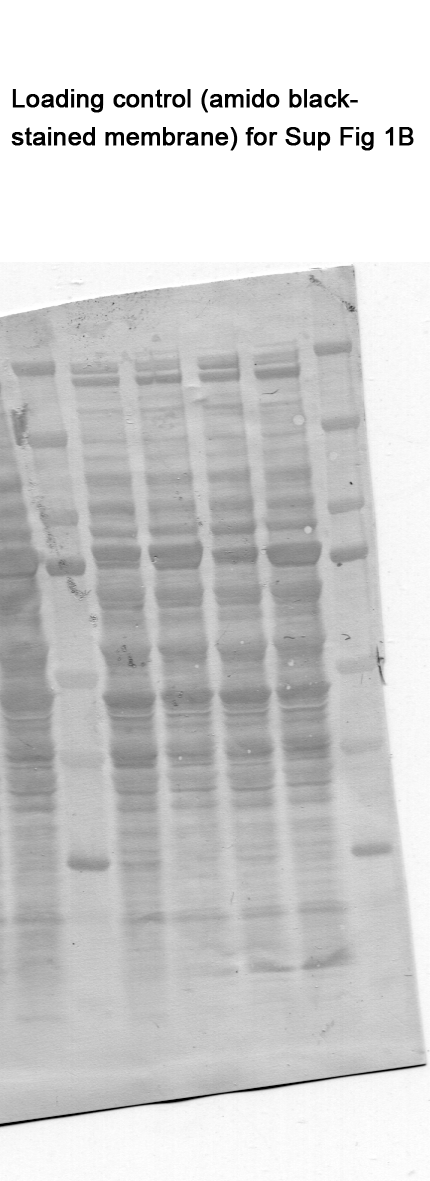

Supplement: Supplementary file 4 — Source Data [file 41467_2023_38230_MOESM4_ESM.zip › Source Data files/Uncropped blots/Uncropped loading control SupFig1B.tif]

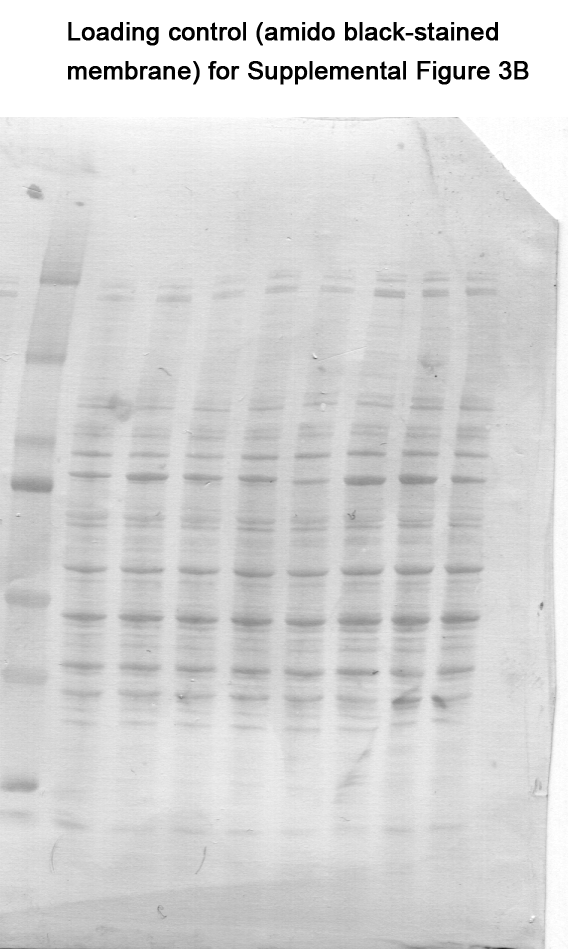

Supplement: Supplementary file 4 — Source Data [file 41467_2023_38230_MOESM4_ESM.zip › Source Data files/Uncropped blots/Uncropped loading control SupFig3B.tif]

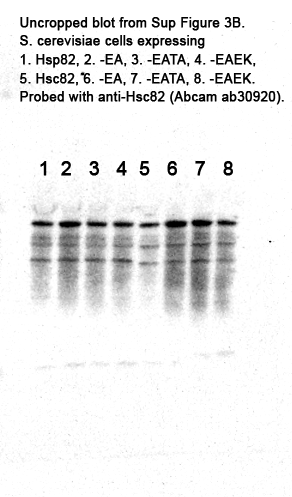

Supplement: Supplementary file 4 — Source Data [file 41467_2023_38230_MOESM4_ESM.zip › Source Data files/Uncropped blots/Uncropped blot SupFig3B.tif]

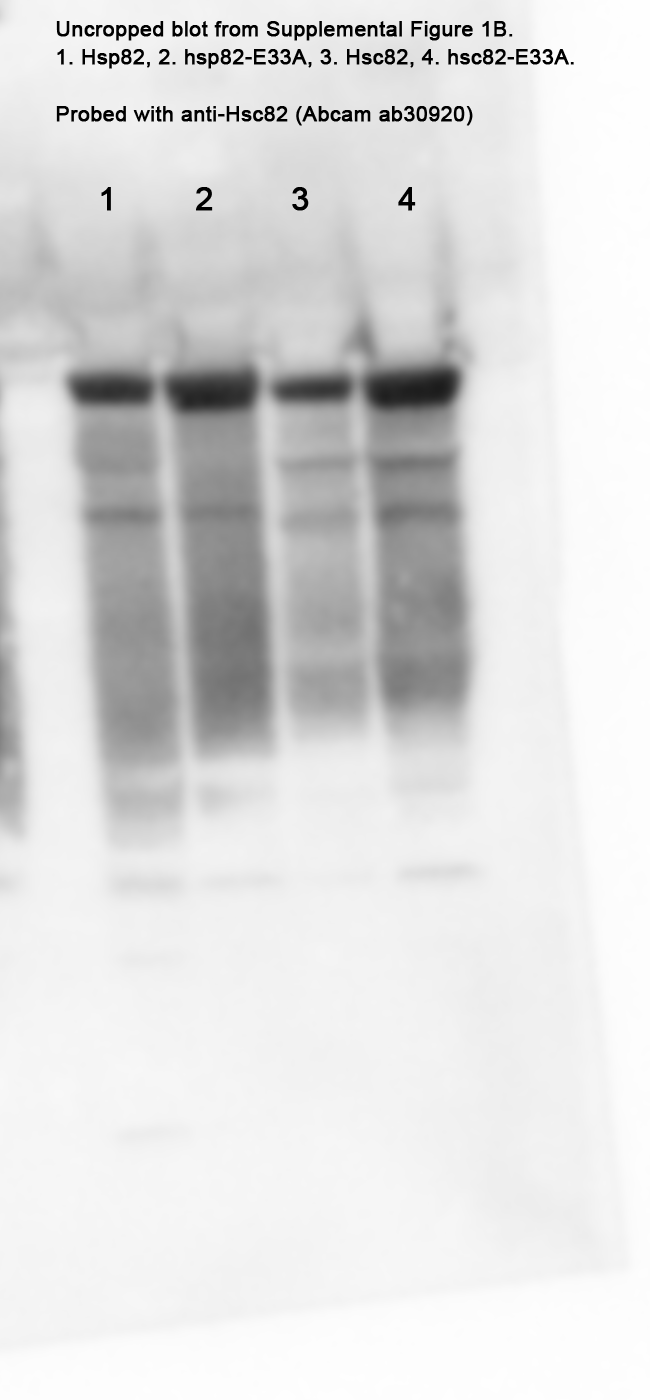

Supplement: Supplementary file 4 — Source Data [file 41467_2023_38230_MOESM4_ESM.zip › Source Data files/Uncropped blots/Uncropped blot SupFig1B.tif]
